# The Relationship between Acculturation and Experimental Pain Sensitivity in Asian Americans with Knee Osteoarthritis

**DOI:** 10.1155/2018/9128015

**Published:** 2018-11-29

**Authors:** Hyochol Ahn, Setor K. Sorkpor, Miyong Kim, Hongyu Miao, Chengxue Zhong, Jing Wang, Debra Lyon, Roger B. Fillingim

**Affiliations:** ^1^Cizik School of Nursing, University of Texas Health Science Center at Houston, Houston, Texas, USA; ^2^School of Nursing, University of Texas, Austin, Texas, USA; ^3^School of Public Health, University of Texas Health Science Center at Houston, Houston, Texas, USA; ^4^School of Nursing, University of Texas Health Science Center at San Antonio, San Antonio, Texas, USA; ^5^College of Nursing, University of Florida, Gainesville, Florida, USA; ^6^University of Florida, Pain Research and Intervention Center of Excellence, Gainesville, Florida, USA

## Abstract

Multiple studies in healthy populations and clinical samples have shown that ethnic minorities have greater pain sensitivity than their majority counterparts. Acculturation is speculated to be one of the sociocultural factors contributing to pain sensitivity since cultural beliefs and practices can influence the way patients perceive and respond to pain. However, the relationship of acculturation to pain sensitivity in minority populations remains poorly understood. Therefore, in this cross-sectional study, we examined the relationship between acculturation and experimental pain sensitivity in 50 Asian Americans residing in North Central Florida with knee osteoarthritis pain. The Suinn-Lew Asian Self Identity Acculturation Scale was used to assess acculturation, and multimodal quantitative sensory testing was performed to measure experimental sensitivity, including heat pain tolerance, pressure pain threshold, and punctate mechanical pain. Descriptive and regression analyses were performed. Participants' mean age was 55.7 years, and about half of this sample were Korean American (56%). The participants had lived in the United States for 21 years on average. Regression analyses indicated that lower acculturation to American culture may contribute to greater experimental pain sensitivity. Asian Americans who were more acculturated to the American culture had higher heat pain tolerance (beta = 0.61, *P*=0.01), higher pressure pain threshold (beta = 0.59, *P*=0.02), and lower ratings of punctate mechanical pain (beta = −0.70, *P* < 0.01). These findings add to the literature regarding sociocultural factors associated with pain in Asian Americans; additional research with a larger and more diverse sample of Asian Americans is warranted for cross-validation.

## 1. Introduction

Chronic pain affects 100 million people in the United States and produces annual costs of up to $635 billion [1, 2]. Arthritis is one of the leading causes of pain, impairments of activities in daily life, and disability in people aged 45 and above [3, 4]. Knee osteoarthritis (OA) is the most common of the arthritic conditions [4–6], and racial/ethnic group differences in knee OA prevalence and its adverse effects have been widely documented [7]. Indeed, some evidence shows that the prevalence of knee OA is higher by 16–75% in Asian females than age-matched White females [7–9].

Ethnic group disparities in health outcomes are well documented but limited to one or two ethnic minority groups, who have poor outcomes in comparison with Whites. Despite several studies demonstrating ethnic differences in pain [10, 11], little research has examined pain experiences in Asians Americans. While it is generally believed that Asian Americans are less sensitive to pain or that their pain experiences do not differ from those of Whites [12], recent studies have reported greater experimental pain sensitivity in Asians than in Whites [13–18].

The National Academy of Medicine (previously Institute of Medicine) highlights the importance of research that promotes health equity and eliminates health disparities by investigating the interplay of multidimensional determinants of health and wellness for all populations and that applies this knowledge to the design of personalized interventions [2]. Because the biopsychosocial model of pain hypothesizes that pain is dynamically affected by biological, psychological, and sociocultural factors [19], it is important to understand the sociocultural factors that impact health disparities in pain among Asian Americans. Acculturation, an essential cultural factor that significantly impacts Asian immigrants' health, is defined as the process of cultural adaptation that takes place when an individual has a prolonged exposure to a new culture and is speculated to affect pain sensitivity, since cultural beliefs and practices can influence the way patients perceive and respond to pain [20, 21]. The literature has indicated that ethnic minority groups with a relatively shorter history of immigration may develop more sensitivity to pain because they must deal with the stresses of acculturation in their new environment [22, 23]. Although Asian Americans were the fastest growing ethnic group in the United States between 2000 and 2010 [24], the previous studies have not explored the contribution of acculturation to pain sensitivity in this population. Therefore, the aim of this study was to examine the relationship between acculturation and experimental pain sensitivity in Asian Americans with knee OA pain. We hypothesized that lower levels of acculturation to the American culture in Asian Americans may contribute to greater experimental pain sensitivity.

## 2. Materials and Methods

### 2.1. Participants

Fifty Asian Americans aged 45 to 85 with symptomatic knee OA pain were recruited in North Central Florida via posted fliers and an e-mail advertisement sent to Asian community LISTSERVS. The individuals were provided information about the study, and interested candidates underwent screening for eligibility. Inclusion criteria included Asian Americans who could speak and read English, 45 to 85 years of age, and self-reported knee OA pain. According to American College of Rheumatology criteria [25], participants should meet at least 3 of 6 criteria, including age >50 years, stiffness <30 minutes, crepitus, bony tenderness, bony enlargement, and no palpable warmth. One of our study team members who is a nationally certified nurse practitioner assessed participants using these inclusion criteria. Exclusion criteria included serious medical illness such as heart failure or a history of acute myocardial infarction, peripheral neuropathy, systemic rheumatic disorders, daily opioid use, cognitive impairment, or hospitalization within the preceding year for psychiatric illness. All procedures were approved by the institutional review board at the University of Florida before commencement, and all patients gave oral and written informed consent prior to participation.

### 2.2. Measures

#### 2.2.1. Acculturation

The Suinn-Lew Asian Self Identity Acculturation (SL-ASIA) Scale in English [26] was used to measure acculturation in Asian Americans. This scale consists of 21 items that measure the degree of acculturation in friendship, language, behaviors, and diet. Each item on the scale is rated from 1 to 5; higher scores indicate greater acculturation to American culture. The 21 items were averaged to generate final acculturation scores that reflected the overall level of acculturation. The SL-ASIA has been reported to have good internal consistency (Cronbach's *α* = 0.88–0.90) and has been used widely in research involving Asian Americans with a wide range of ages, including older adults [27–29].

#### 2.2.2. Experimental Pain Sensitivity

Heat pain tolerance was measured on both the index knee and the ipsilateral ventral forearm with contact heat stimuli delivered via a Medoc Pathway Neurosensory Analyzer (Ramat Yishai, Israel) using an ascending method of limits. The temperature began at 32°C and increased at a rate of 0.5°C per second until participants responded by pressing a button on a handheld device when they “no longer feel able to tolerate the pain.” Heat was applied to each of the two locations three times, and the temperatures of the three individual trials at each body site were averaged to generate heat pain tolerance at each site. We assessed heat pain tolerance, because tolerance is thought to reflect the affective-motivational dimension.

Pressure pain thresholds were measured by a handheld Medoc digital pressure algometer (Algomed) at a constant rate of 30 kPa per second at 5 sites: the medial aspect of the index knee, lateral aspect of the index knee, ipsilateral quadriceps, trapezius, and epicondyle. The order of testing sites was randomized and counterbalanced. To assess pressure pain thresholds, participants were instructed to press the button when the sensation “first becomes painful.” Pressure was applied to each of the five locations three times, and the results of the three individual trials at each body site were averaged to generate pressure pain threshold at each site.

Punctate pain sensitivity was measured using a calibrated nylon monofilament delivering a target force of 300 g applied on the index patella as well as the back of the ipsilateral hand. Participants provided numerical pain intensity ratings on a scale from 0 (no pain sensation) to 100 (the most intense pain sensation imaginable) after 10 contacts at a rate of 1 contact per second at each of the two locations. This procedure was performed twice at each site, and the results of the two individual trials at each body site were averaged to generate punctate mechanical pain at each site.

### 2.3. Statistical Analysis

All analyses were conducted with SAS version 9.4. Descriptive statistics appropriate for the level of measurement were used to validate values and evaluate missing and variable distributions. For purposes of variable reduction, we created composite measures of pain sensitivity. First, the normality of data distribution was checked by using the Shapiro–Wilk test for the four pain measurements. Only the normality of pressure pain threshold did not hold; therefore, the Box-Cox transformation was applied on raw data of pressure pain threshold. After the Box-Cox transformation on pressure pain threshold, *z*-scores were computed for heat pain tolerance measurements at the arm and knee; pressure pain threshold measures at the medial knee, lateral knee, quadriceps, trapezius, and epicondyle; and punctate pain measurements at the patella and hand. Then, the *z*-scores for each pain measure were averaged across body sites to derive an overall measure of heat pain tolerance, pressure pain threshold, and punctate pain. We created *z*-scores for each pain modality for variable reduction, as in prior research. Specifically, previous factor analytic studies have demonstrated that laboratory pain measures typically aggregate within pain modality. That is, factor analytic solutions generally reveal a heat pain factor, a pressure pain factor, and so on [30–32]. Thus, investigators have commonly computed pain index scores by averaging *z*-scores within pain modalities [33, 34]. This allows variable reduction, while also placing all pain measures on a common metric without altering the distributional characteristics of the underlying data. These *z*-scores were used as outcomes to analyze the relationship between pain sensitivity and acculturation. Gender, age, acculturation, and BMI were used to fit separate linear regression models for each experimental pain measure. SAS PROC GLM was employed to estimate the parameters after fixing the age, BMI, and gender in our model.

## 3. Results

### 3.1. Demographic Characteristics

Fifty Asian Americans were enrolled for this study (see Table 1 for details). About half of this sample was Korean Americans (56%), mean age was 55.66 (standard deviation (SD) = 7.81), and the participants had lived in the United States for about 21 years (SD = 15.15). The mean BMI in the sample was 24.18 (SD = 3.03). Participants had an average SL-ASIA score of 2.18 (SD = 0.54), indicating an average of low acculturation to the American culture in the study sample.

### 3.2. Relationship between Acculturation and Experimental Pain Sensitivity

There was a signification relationship between acculturation and all three pain sensitivity measures (Table 2). Asian Americans who were more acculturated to the American culture had higher heat pain tolerance (beta = 0.61, *P*=0.01), higher pressure pain threshold (beta = 0.59, *P*=0.02), and lower rating of punctate mechanical pain (beta = −0.70, *P* < 0.01). In addition, male Asian Americans in the United States had greater heat pain tolerance (beta = 0.87, *P* < 0.01), greater pressure pain threshold (beta = 0.79, *P* < 0.01), and lower rating of punctate mechanical pain (beta = −0.52, *P*=0.05) than did female Asian Americans.

## 4. Discussion

In this study to identify the relationship between acculturation and experimental pain sensitivity among Asian Americans, the fastest growing ethnic group in the United States [6], we found that participants with higher acculturation to American culture had lower experimental pain sensitivity (i.e., higher heat pain tolerance, higher pressure pain threshold, and lower punctate pain). We also found no statistically significant interaction between acculturation and gender: male participants reported a higher tolerance and pain threshold than did female participants, regardless of acculturation.

The findings of the present study are consistent with previous evidence showing that individuals with low acculturation to their host culture experience heightened pain. For example, Edrington et al. [35] reported that lower acculturation to American culture was associated with higher pain intensity and pain interference among Asian Americans with cancer. Similarly, Chan et al. [36] reported that first-generation Asian Americans showed greater cold pain sensitivity than European Americans, while second-generation Asian Americans did not differ from the European American group. Also, Palmer et al. [37] found a negative association between acculturation to British culture and prevalence of widespread pain among Asians in the United Kingdom.

The exact mechanisms underlying the relationship between acculturation and pain are unclear, but one possible explanation for the amplified pain sensitivity in relation to low acculturation may be the result of stress arising from conflict between the heritage culture and host culture [17, 38]. These negative experiences may diminish one's sense of self-control, which in turn may lead to higher pain sensitivity. Another possibility is that lower acculturation may increase discriminatory treatment of minorities, which represents a potentially potent chronic stress exposure. Also, lower acculturation could be associated with greater disparities in provision of healthcare [39]. Hence, Asian Americans with lower acculturation may have less familiarization with the language, food, customs, social norms, and values of American culture, which likely produces cross-cultural stresses and thereby increases sensitivity to pain.

This study's results should be interpreted in light of several limitations. First, the sample of 50 Asian Americans originated from five different countries, with about half from Korea. Our sample, therefore, was not truly representative of the Asian American population. Second, Asian Americans in our study were limited to those who could speak and read English, which might have excluded people in the extremely low acculturation spectrum and therefore imposed some interference challenges of findings. It is reasonable to speculate that the magnitude of relationship between acculturation and pain sensitivity might be even greater in non-English-speaking individuals in the United States. Third, we did not consider all confounding variables, but we did statistically control for age, gender, and BMI. Given the small sample size, confounding variables were restricted to pain-related individual factors. Fourth, the data collection was based on a cross-sectional study design, consequently limiting our ability to draw causal inferences.

Future studies conducted with a larger and more heterogeneous sample are needed to validate these findings. Nonetheless, the following clinical implications warrant consideration. Healthcare providers should proactively assess multiple dimensions of pain, including cultural aspects, in Asian American patients. In addition, there is a significant need for culturally sensitive pain interventions to improve pain management in this vulnerable population.

The findings of the present study lay the groundwork for further research to investigate the mechanisms by which acculturation influences pain sensitivity. First, future studies with larger samples could address geographic location of original home country, because there may be subgroup differences among Asian Americans. A more heterogeneous sample would provide a better representative sample allowing for between-group estimations of differences in pain intensity rating in relation to acculturation. Second, future studies are needed to investigate cultural beliefs and values that reduce or heighten pain sensitivity. Finally, future studies that incorporate additional biological and psychological measures are needed to elucidate underlying mechanisms.

## 5. Conclusion

The relationship between acculturation and experimental pain sensitivity in older adults with chronic pain has rarely been examined. The results from our study suggest that lower acculturation to American culture may be associated with heightened pain sensitivity among ethnic minorities in the United States. The present study adds to the growing literature that acculturation has an influence on pain sensitivity. Further investigation is needed to determine the role of specific cultural factors in heightened pain and to ensure that ethnic group disparities in pain are ameliorated via programs directed at promoting acculturation among Asian Americans.

## Figures and Tables

**Table 1:** Demographic and clinical characteristics of participants.

Characteristics	Total
Age (year), mean (SD)	55.66 (7.81)
BMI, (kg/m^2^), mean (SD)	24.18 (3.03)
Gender, *n* (%)	
Male	19 (38)
Female	31 (62)
Original country, *n* (%)	
Korea	28 (56)
China	9 (18)
Japan	7 (14)
Filipino	5 (10)
India	1 (2)
Length of stay at the USA (year), mean (SD)	21.38 (15.15)
Education, *n* (%)	
Less than high school	1 (2)
High school	7 (14)
Some college	8 (16)
Bachelor's degree	14 (28)
Graduate degree	20 (40)
Heat pain tolerance (°C), mean (SD)^†^	41.84 (3.92)
Pressure pain threshold (kPa), mean (SD)^†^	181. 23 (85.01)
Punctate pain, mean (SD)^†^	40.43 (23.69)
SL-AISA, mean (SD)	2.18 (0.55)

*Note.* BMI, body mass index. Data are presented as mean (standard deviation) or number (percentage). Measured on the SL-ASIA, total scores range from 1 to 5, with higher scores indicating more acculturation to American culture. ^†^Overall average score.

**Table 2:** Results of regression model.

Variable	Beta	Standard error	*t* value	*P* value
Heat pain tolerance^*∗*^				
Acculturation	0.61	0.23	2.69	0.01
Age	−0.01	0.02	−0.70	0.49
Male	0.87	0.25	3.45	<0.01
BMI	0.03	0.04	0.84	0.40

Pressure pain threshold^*∗*^				
Acculturation	0.59	0.24	2.48	0.02
Age	0.01	0.02	0.82	0.41
Male	0.79	0.27	2.96	<0.01
BMI	0.05	0.04	1.16	0.25

Punctate pain^*∗*^				
Acculturation	−0.70	0.23	−2.99	<0.01
Age	0.01	0.02	0.70	0.49
Male	−0.52	0.26	−1.97	0.05
BMI	−0.02	0.04	−0.38	0.71

*Note.* BMI, body mass index. R-square: heat pain tolerance = 0.32, pressure pain threshold = 0.25, punctate pain = 0.22. ^*∗*^Average *z*-score.

## Data Availability

The data used to support the findings of this study are available from the corresponding author upon request.
